# Sinus Arrest Related to Dexmedetomidine Infusion in an Infant; a Case Report and Review of Current Literature

**DOI:** 10.1177/08971900231193558

**Published:** 2023-08-04

**Authors:** Alexandra Dennee, Thomas P. Fogarty, Taylor S. Howard, Ryan Brandon Hunter

**Affiliations:** 1 3989 Department of Pediatrics, Baylor College of Medicine, Houston, TX, USA; 2 Department of Pediatrics, Section of Critical Care Medicine, 3989 Baylor College of Medicine, Houston, TX, USA; 3 Department of Pediatrics, Section of Cardiology, Baylor College of Medicine, Houston, TX, USA

**Keywords:** dexmedetomidine, sinus arrest, asystole, sedation, pediatrics

## Abstract

**Background:** Dexmedetomidine, an alpha 2 agonist, has emerged as a desirable sedative agent in the pediatric intensive care unit due to its minimal effect on respiratory status and reduction in delirium. Bradycardia and hypotension are common side effects, however there are emerging reports of more serious cardiovascular events, including sinus arrest and asystole. These case reports have been attributed to high vagal tone or underlying cardiac conduction dysfunction. **Objectives:** To describe the development of sinus arrest during sedation with dexmedetomidine in a patient without clinical features of high vagal tone, underlying cardiac conduction dysfunction, or intervening episodes of bradycardia. **Case Presentation:** An 11 month-old patient requiring sedation during mechanical ventilation for acute respiratory failure secondary to Adenovirus. To facilitate sedation, a dexmedetomidine infusion was initiated at .5 mcg/kg/hr and increased to maximum 1 mcg/kg/hr. Within 8 hours of initiating therapy, the patient had three episodes of sinus arrest. There was no intervening bradycardia between episodes and no further episodes occurred following discontinuation of dexmedetomidine. The patient did not have any clinical features associated with high vagal tone or underlying cardiac conduction dysfunction. **Conclusions:** As result of these findings, understanding risk factors for bradycardia, or more serious hemodynamic instability with dexmedetomidine infusions, is important to help identify high risk patients and weigh the associated risks and benefits of its administration.

## Introduction

Dexmedetomidine is a highly selective, centrally acting alpha 2 agonist with anxiolytic, sedative, and analgesic effects.^1^ It has emerged as a desirable sedative agent in pediatric intensive care due to its minimal effect on ventilatory drive and reduction in delirium when compared to benzodiazepines.^1,2^ Due to its sympatholytic effects, common side effects include bradycardia and hypotension. Studies report a variable incidence of bradycardia between 0-42% among patients receiving dexmedetomidine.^3,4^ There are emerging case reports of more serious cardiovascular events, including sinus arrest and asystole, in the pediatric population.^5-11^ Most of these case reports have been attributed to high vagal tone, rapid medication infusion, concurrent administration of a negative chronotropic agent, or underlying cardiac conduction dysfunction. All reported events were preceded by severe bradycardia or were associated with a clinical event precipitating a strong vagal response (eg bearing down, stooling, agitation, hypoxia, stimulation of the carina). We present a case of a pediatric patient who developed multiple episodes of sinus arrest associated with a dexmedetomidine infusion without clinical features associated with high vagal tone or intervening bradycardia between events in the pediatric intensive care unit (PICU).

## Case Presentation

**Figure 1.**
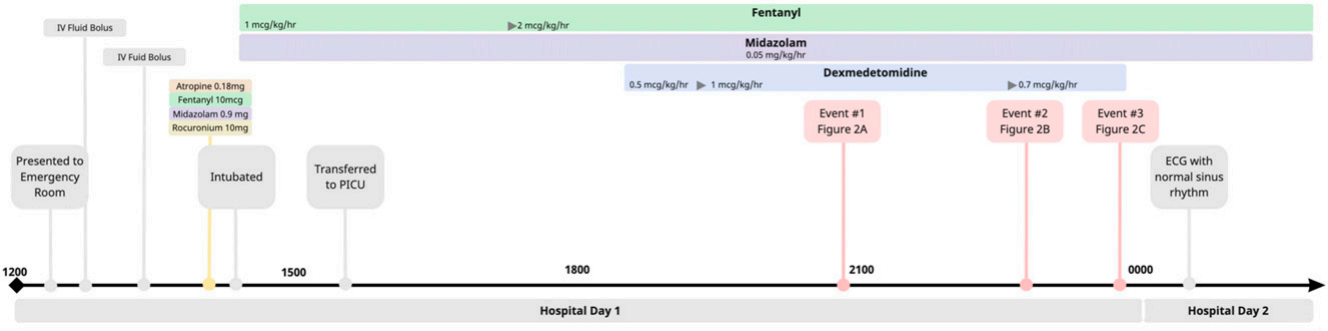
Timeline reviewing clinical course including medications administered and sinus pause events.

An 11 month-old previously healthy female presented in acute hypoxemic respiratory failure secondary to Adenovirus bronchiolitis requiring mechanical ventilation. To facilitate sedation, a dexmedetomidine infusion was initiated at .5 mcg/kg/hr and increased to a maximum dose of 1 mcg/kg/hr. During this time, there was a decline in average heart rate from 175 to 135 but was still well within a normal range for age (Figure 1).

**Figure 2.**
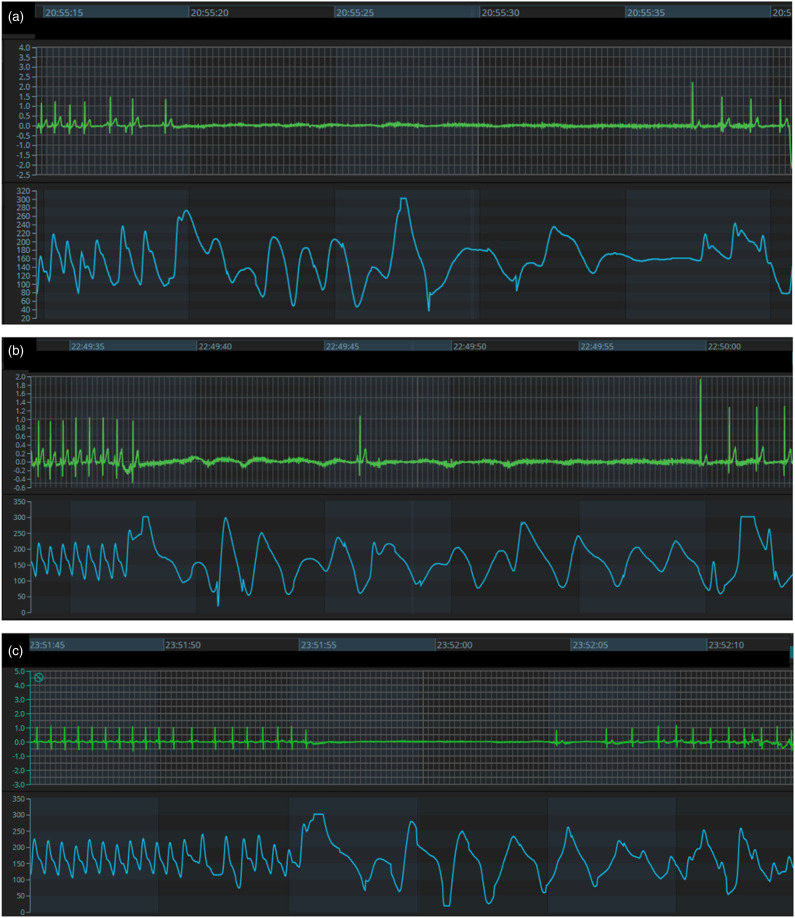
Displays the ECG and SpO2 tracing during each of the three episodes of sinus arrest (A-C). Note that there is mild bradycardia prior to sinus arrest in the first event, but a normal sinus rate preceding the latter two. The patient did not have any episodes of bradycardia <110 beats per minute in the intervening period between any of these episodes.

Within 8 hours of initiating therapy, she was noted to have three episodes of sinus arrest. The first episode was 18 seconds with resumption of junctional and then sinus rhythm. The second arrest was 9 seconds with a single junctional escape beat followed by another 13 second pause, with eventual resumption of a regular junctional escape and then normal sinus rhythm. A third episode was 10 seconds in duration. These episodes occurred spontaneously in the setting of an intervening sinus rate >120 bpm throughout (Figure 2). Dexmedetomidine was running at 1 mcg/kg/hr during the first two episodes and .7 mcg/kg/hr during the third. Each episode self-resolved prior to initiation of cardiopulmonary resuscitation. Several hours after the events, the patient required low-dose epinephrine for ∼24 hours for mild hypotension attributed to compensated septic shock; of note, this hypotension was not accompanied by bradycardia or other rhythm disturbance. After discontinuation of dexmedetomidine, there was no reoccurrence of sinus arrest.

These events occurred with the patient comfortably sedated and without clinical features usually associated with high vagal tone such as increased agitation, ventilator dyssynchrony, hypoxia, coughing, or straining with bowel movement. A chest radiograph was obtained and confirmed placement of endotracheal tube within the upper thoracic trachea, and the patient had not received endotracheal tube suctioning prior to any episode, making carina stimulation an unlikely contributor. Dexmedetomidine was co-administered with fentanyl, midazolam and ceftriaxone, but the patient did not receive corticosteroids or other medications associated with negative chronotropic effects. Laboratory evaluation immediately following the events revealed electrolytes (potassium, magnesium, calcium) within normal limits. An echocardiogram demonstrated normal biventricular systolic function and structural anatomy. She had no other known risk factors for sinus node dysfunction, including congenital heart disease, known cardiac conduction deficits, heterotaxy, family history of cardiac disease, or signs of viral myocarditis.

The remainder of her hospitalization was remarkable for sepsis secondary to *Haemophilus influenzae* pneumonia that resolved with appropriate antimicrobial treatment. She was extubated to non-invasive positive pressure ventilation and subsequently weaned to room air. Throughout the remainder of her hospitalization, she had a sinus rhythm with normal heart rate variation. At time of discharge, she was doing well on room air and tolerating her home diet.

## Discussion

We report an infant who experienced three prolonged but self-resolving episodes of sinus arrest while receiving dexmedetomidine for sedation, without recurrence after discontinuation. The case is unique given the duration and characteristics of the episodes. Although the episodes are reminiscent of elevated vagal tone, there was no evidence of sinus bradycardia in between the episodes nor any common clinical precipitants of vagal excess which are commonly reported.

The mechanism by which dexmedetomidine causes sinus and AV node dysfunction remains uncertain. Literature shows dexmedetomidine significantly depresses the sinus and AV node function in pediatric patients as evidenced by an increase in sinus cycle length, sinus node recovery time, PR interval, and Wenckebach cycle length.^12^ A recent meta-analysis of side effects in patients receiving dexmedetomidine for ≥24 hours reported a pooled prevalence of bradycardia at 2.6% and hypotension at 6.1%; the authors defined these side effects as hemodynamic changes that required intervention.^4^ When bradycardia or hypotension occurs, it is usually within 12 hours of initiation and are reversible with discontinuation of the drug.^4^

Excessive vagal response is a common cause of bradycardia in the PICU and could precipitate severe bradycardia or sinus arrest when superimposed on the already-depressed sinus rate. Common causes of vagal excess include nasopharyngeal or esophageal stimulation (eg nasogastric or endotracheal tube placement), carina stimulation (eg from suctioning or a low-lying endotracheal tube), breath-holding spells, gastroesophageal reflux, stooling, coughing, or ventilator dyssynchrony. In our case, the cause of bradycardia possibly had a vagal contribution, but the only identifiable trigger was the well-positioned endotracheal tube, which would act as a static vagal stimulant. All prior case reports have occurred in patients with preceding bradycardia or clear clinical events that led to excessive vagal tone (eg coughing, agitation, suctioning). Many of these patients were also receiving medications, had history of cardiac conduction defects, or were being cooled, contributing to negative chronotropy (Table 1).^5-11^

**Table 1.** Reports of Sinus Pauses and Asystole Associated with Dexmedetomidine in Pediatric Patients. LTR = laryngotracheal reconstruction. VFib = ventricular fibrillation. RSV = Respiratory Syncytial Virus.

Authors	Clinical Description	Dose of Dexmedetomidine (mcg/kg/hr)	Preceding Bradycardia or precipitating event	Duration of Sinus Pause or Asystole	Outcome, Corrective Action
Berkenbosch et al, 2003^5^	5 week-old with atrioventricular septal defect intubated for RSV bronchiolitis, on digoxin	.44	Progressive bradycardia	N/A	Resolved with discontinuation of dexmedetomidine
Sharron et al, 2009^6^	3 month-old status post LTR, intubated	1	Progressive bradycardia	6 seconds	Self-resolved, sinus rhythm
	13 month-old status post VFib arrest, loaded with amiodarone, cooled to 34C, intubated	1	Endotracheal tube suctioning	9 seconds	Self-resolved, ventricular escape then sinus rhythm
	2 month-old following cricoid split, intubated	1	Progressive bradycardia	5 seconds	Self-resolved, sinus rhythm
Zhang et al, 2010	18 year-old with cystic fibrosis and double lung transplant status post fundoplication, intubated	.4 – .8	Coughing spasm, bradycardia	5 episodes, max duration of 10 seconds	Self-resolved, sinus rhythm
Shepard et al, 2011^7,8^	3 year-old with repaired tetralogy of Fallot and dual chamber pacemaker for heart block status post mitral valve replacement, intubated	.6	Sinus and junctional arrest with failure of atrial capture by temporary or permanent pacemaker	Prolonged	Resolved with discontinuation of dexmedetomidine
Banc-Husu et al, 2021^9^	9 month-old with biliary atresia status post liver transplant, intubated	1	Bradycardia	4 seconds	Self-resolved
		1	Endotracheal tube suctioning, bradycardia	7 seconds	Brief chest compressions and bag-mask ventilation
Kojima et al, 2021^10^	2 month-old receiving noninvasive ventilation for RSV bronchiolitis	.25	Bradycardia	4 seconds	Self-resolved, junctional escape then sinus rhythm
	2 month-old receiving noninvasive ventilation for RSV bronchiolitis	.5	Bradycardia	10 seconds	Self-resolved, sinus rhythm
Lichtsinn et al, 2021^11^	8 week-old receiving noninvasive ventilation for RSV bronchiolitis	.7	Intermittent bradycardia, coughing spells	6 seconds	Self-resolved
	27 month-old with pulmonary hypertension, status post repeat repair of diaphragmatic hernia, intubated	1.1	Endotracheal tube suctioning	15 seconds followed by bradycardia	2 minutes of CPR

Dexmedetomidine received FDA approval for sedation in 1999, and since that time has become widely used in adult and pediatric critical care. The Clinical Practice Guidelines for the Prevention and Management of Pain, Agitation/Sedation, Delirium, Immobility, and Sleep Disruption in Adult Patients in the ICU suggest using propofol or dexmedetomidine over benzodiazepines for sedation in critically ill, mechanically ventilated adults due to a shortened time to extubation.^13^ For mechanically ventilated adults, the guidelines also suggest using dexmedetomidine for delirium where agitation is precluding weaning or extubation.^13^ Adult studies frequently note increased incidence of bradycardia and hypotension (and possibility of greater self-extubation).^14^ The recent Intensive Care Medicine Rapid Practice Guideline recommends that dexmedetomidine should be used over other sedative agents when desirable effects of reduced delirium and duration of mechanical ventilation are valued over undesirable effects of hypotension and bradycardia.^14^

Although only FDA approved in the adult population, dexmedetomidine use has also become more prevalent in the pediatric intensive care unit (PICU).^4^ Despite its increased use, there are few prospective and randomized studies analyzing dexmedetomidine use for ≥24 hours in the PICU.^4,15,16^ One notable recent study was the PROSDEX study, a prospective multicenter study evaluating the efficacy of dexmedetomidine for prolonged sedation (≥24 hours) in 163 critically ill pediatric patients using validated clinical scores to evaluate level of sedation, withdrawal, and delirium. After 24 hours of initiation, dexmedetomidine was associated with improved patient comfort, withdrawal, and delirium scores, and reduced dosages of alternative sedative agents (benzodiazepines, opioids, propofol, and ketamine).^15^ Bradycardia was reported in 27% of patients and hypotension in 11%. For hemodynamic adverse effects that required intervention (9% of total patients), a dose reduction was the most common intervention.^15^ Banasch et al^16^ demonstrated an association between adverse effects with younger age and longer dexmedetomidine duration. A randomized controlled trial is currently ongoing to evaluate the efficacy and safety of dexmedetomidine for prevention of withdrawal syndrome in the pediatric intensive care unit.^17^

The 2022 Society of Critical Care Medicine Guidelines on Prevention and Management of Pain, Agitation, Neuromuscular Blockade, and Delirium in Critically Ill Pediatric Patients With Consideration of the ICU Environment Early Mobility (PANDEM) Guidelines now recommend the use of dexmedetomidine as the primary agent for critically ill post-operative cardiac patients with expected early extubation, with a conditional recommendation regarding its use in this setting to prevent tachyarrhythmias. For the general PICU population, the guidelines recommend minimizing benzodiazepine exposure.^18^ Although this is not a positive recommendation for dexmedetomidine use, it is commonly favored given the significant drawbacks of alternative agents (propofol, ketamine, barbiturates) has seen success in ICU sedation practices due to its associated reduction in benzodiazepine exposure and reduction in delirium. Despite these advantages, in the pediatric population where FDA approval is lacking, there are considerable hemodynamic risks associated with the use of the medication, with 5-10% of patients experiencing a bradycardia or hypotension that requires intervention. Our case is unique given the duration of events and lack of strong precipitating factors outside of dexmedetomidine use. In addition to previous pediatric case reports in the literature, the authors suggest that dexmedetomidine should be used with careful weight of the associated risks and benefits.

## References

[1] Tobias JD . Dexmedetomidine: Applications in pediatric critical care and pediatric anesthesiology. Pediatr Crit Care Med. 2007;8(2):115-131. doi:10.1097/01.PCC.0000257100.31779.41 17273114

[2] Lee S . Dexmedetomidine: Present and future directions. Korean J Anesthesiol. 2019;72(4):323-330. doi:10.4097/kja.19259 31220910 PMC6676029

[3] Riker RR . Dexmedetomidine vs midazolam for sedation of critically Ill PatientsA randomized trial. JAMA. 2009;301(5):489. doi:10.1001/jama.2009.56 19188334

[4] Daverio M Sperotto F Zanetto L , et al. Dexmedetomidine for prolonged sedation in the PICU: A systematic review and meta-Analysis. Pediatr Crit Care Med. 2020;21(7):e467. doi:10.1097/PCC.0000000000002325 32453924

[5] Berkenbosch JW Tobias JD . Development of bradycardia during sedation with dexmedetomidine in an infant concurrently receiving digoxin. Pediatr Crit Care Med. 2003;4(2):203-205. doi:10.1097/01.PCC.0000059737.86673.28 12749653

[6] Sharron M Klugman D Moak J Dean N . Dexmedetomidine induced bradycardia progressing to sinus arrest: A series of three patients. American Academy of Pediatrics National Conference and Exhibition, Washington, DC, USA, 2009.

[7] Zhang X Schmidt U Wain JC Bigatello L . Bradycardia leading to asystole during dexmedetomidine infusion in an 18 year-old double-lung transplant recipient. J Clin Anesth. 2010;22(1):45-49. doi:10.1016/j.jclinane.2009.06.002 20206851

[8] Shepard SM Tejman-Yarden S Khanna S Davis CK Batra AS . Dexmedetomidine-related atrial standstill and loss of capture in a pediatric patient after congenital heart surgery. Crit Care Med. 2011;39(1):187-189. doi:10.1097/CCM.0b013e3181feb4b3 20959781

[9] Banc‐Husu AM Badke CM Sanchez Pinto LN Alonso EM . Dexmedetomidine leading to profound bradycardia in a pediatric liver transplant recipient. Pediatr Transplant. 2021;25(5). doi:10.1111/petr.13895 33118274

[10] Kojima H Tanaka R Iwamoto Y Ishido H Sakurai Y Masutani S . Markedly long pause due to sinus arrest during dexmedetomidine use and nasal continuous positive airway pressure in two infants with respiratory syncytial virus infection. Journal of Cardiology Cases. 2021;23(1):10-12. doi:10.1016/j.jccase.2020.08.012 33437332 PMC7783580

[11] Lichtsinn K Sehgal I Wilson A . Asystole in 2 pediatric patients during dexmedetomidine infusion. Journal of Pharmacy Practice. 2021;24:089719002110271. doi:10.1177/08971900211027133 34165021

[12] Hammer GB Drover DR Cao H , et al. The effects of dexmedetomidine on cardiac electrophysiology in children. Anesth Analg. 2008;106(1):79-83. doi:10.1213/01.ane.0000297421.92857.4e 18165557

[13] Devlin JW Skrobik Y Gélinas C , et al. Clinical practice guidelines for the prevention and management of pain, agitation/sedation, delirium, immobility, and sleep disruption in adult patients in the ICU. Crit Care Med. 2018;46(9):e825-e873. doi:10.1097/CCM.0000000000003299 30113379

[14] Møller MH Alhazzani W Lewis K , et al. Use of dexmedetomidine for sedation in mechanically ventilated adult ICU patients: A rapid practice guideline. Intensive Care Med. 2022;48(7):801-810. doi:10.1007/s00134-022-06660-x 35587274

[15] Sperotto F Mondardini MC Dell’Oste C , et al. Efficacy and safety of dexmedetomidine for prolonged sedation in the PICU: A prospective multicenter study (PROSDEX). Pediatr Crit Care Med. 2020;21(7):625-636. doi:10.1097/PCC.0000000000002350 32224830

[16] Banasch HL Dersch-Mills DA Boulter LL Gilfoyle E . Dexmedetomidine use in a pediatric intensive care unit: A retrospective cohort study. Ann Pharmacother. 2018;52(2):133-139. doi:10.1177/1060028017734560 28952341

[17] Mondardini MC Sperotto F Daverio M , et al. Efficacy and safety of dexmedetomidine for prevention of withdrawal syndrome in the pediatric intensive care unit: Protocol for an adaptive, multicenter, randomized, double-blind, placebo-controlled, non-profit clinical trial. Trials. 2019;20(1):710. doi:10.1186/s13063-019-3793-6 31829274 PMC6907190

[18] Smith HAB Besunder JB Betters KA , et al. 2022 society of critical care medicine clinical practice guidelines on prevention and management of pain, agitation, neuromuscular blockade, and delirium in critically Ill pediatric patients with consideration of the ICU environment and early mobility. Pediatr Crit Care Med. 2022;23(2):e74. doi:10.1097/PCC.0000000000002873 35119438

